# Role of Cytokine Combinations on CD4+ T Cell Differentiation, Partial Polarization, and Plasticity: Continuous Network Modeling Approach

**DOI:** 10.3389/fphys.2018.00877

**Published:** 2018-08-02

**Authors:** Mariana E. Martinez-Sanchez, Leonor Huerta, Elena R. Alvarez-Buylla, Carlos Villarreal Luján

**Affiliations:** ^1^Laboratorio Genética Molecular, Epigenética, Desarrollo y Evolución de Plantas, Departamento de Ecología Funcional, Instituto de Ecología, Universidad Nacional Autónoma de México, Mexico City, Mexico; ^2^Centro de Ciencias de la Complejidad, Universidad Nacional Autónoma de México, Mexico City, Mexico; ^3^Laboratorio B108, Departmento de Immunología, Instituto de Investigaciones Biomédicas, Universidad Nacional Autónoma de México, Mexico City, Mexico; ^4^Departamento de Física Cuántica y Fotónica, Instituto de Física, Universidad Nacional Autónoma de México, Mexico City, Mexico

**Keywords:** CD4+ T cells, regulatory network, ODE, heterogeneity, plasticity, micro-environment, cytokines

## Abstract

**Purpose:** We put forward a theoretical and dynamical approach for the semi-quantitative analysis of CD4+ T cell differentiation, the process by which cells with different functions are derived from activated CD4^+^ T naïve lymphocytes in the presence of particular cytokine microenvironments. We explore the system-level mechanisms that underlie CD4+ T plasticity-the conversion of polarized cells to phenotypes different from those originally induced.

**Methods:** In this paper, we extend a previous study based on a Boolean network to a continuous framework. The network includes transcription factors, signaling pathways, as well as autocrine and exogenous cytokines, with interaction rules derived using fuzzy logic.

**Results:** This approach allows us to assess the effect of relative differences in the concentrations and combinations of exogenous and endogenous cytokines, as well as of the expression levels of diverse transcription factors. We found either abrupt or gradual differentiation patterns between observed phenotypes depending on critical concentrations of single or multiple environmental cytokines. Plastic changes induced by environmental cytokines were observed in conditions of partial phenotype polarization in the T helper 1 to T helper 2 transition. On the other hand, the T helper 17 to induced regulatory T-cells transition was highly dependent on cytokine concentrations, with TGFβ playing a prime role.

**Conclusion:** The present approach is useful to further understand the system-level mechanisms underlying observed patterns of CD4^+^ T differentiation and response to changing immunological challenges.

## Introduction

The phenotype of a cell emerges from the feedback between internal regulatory networks and signals from the microenvironment (Murphy and Stockinger, 2010; DuPage and Bluestone, 2016). CD4+ T cells constitute a useful model to evaluate the role of micro-environmental signals on intracellular regulatory networks underlying cell differentiation and plasticity, as the combination and concentration of exogenous cytokines are crucial for CD4+ T cell differentiation and plasticity (Murphy and Stockinger, 2010; DuPage and Bluestone, 2016; Eizenberg-Magar et al., 2017).

CD4+ T cells are part of the adaptive immune response. Naïve CD4+ T cells are activated in response to antigens presented by antigen presenting cells (APC) (Zhu et al., 2010). Depending on the cytokines in the microenvironment, these cells may differentiate into particular subsets. APCs are the main source of cytokines (extrinsic cytokines) initiating an immune response, but they can also be produced by other cells of the organism (Duque and Descoteaux, 2014; Sozzani et al., 2017). Exogenous cytokines bind to the membrane receptors of the cell and activate intracellular signaling pathways. These signals activate or inhibit particular transcription factors integrated in the networks under analysis and promote the production of autocrine cytokines, creating a positive feedback that reinforces the polarization dynamics (Zhu et al., 2010). In addition, autocrine cytokines that can also activate or inhibit other cells of the immune system. It is interesting to note that different cytokines combinations have been shown to have synergistic or antagonistic effects on CD4+ T cell differentiation, and such differential responses may be crucial during immune responses to pathogen attack, modulation of the immune response, or immunopathological conditions (Zhu et al., 2010).

Functional CD4+ T lymphocytes can be grouped into subsets known as Th1, Th2, Th3, Th9, Th17, Treg, Tr1, and Tfh (**Table 1**). It has been documented that Th1 cells require extrinsic IL-12 and IFNγ, they express T-bet and IFNγ (Hsieh et al., 1993; Perez et al., 1995; Szabo et al., 2000, 2003). Th2 cells require extrinsic IL-4 and are stabilized by IL-2, they express GATA3, IL-4, IL-5, and -IL13 (Le Gros et al., 1990; Swain et al., 1990; Cote-Sierra et al., 2004; Ansel et al., 2006; Zheng and Flavell, 1997). Th3 cells require extrinsic TGFβ and express TGFβ (Gol-Ara et al., 2012). Th9 cells require IL-4 and TGFβ, they express IL-9 (Lu et al., 2012; Kaplan, 2013; Schmitt et al., 2014). Th17 cells require extrinsic TGFβ and IL-6, IL-21 or IL-23, they produce RORγt, IL-21, IL-17A, and IL-17F (Ivanov et al., 2006; Veldhoen et al., 2006; Zhou et al., 2007; Korn et al., 2009). Treg cells require extrinsic TGFβ and IL-2, they express Foxp3, TGFβ and in some cases IL-10 (Chen et al., 2003; Hori et al., 2003; Davidson et al., 2007; Zheng et al., 2007). Tr1 cells require extrinsic IL10, expressing IL10 (Roncarolo et al., 2006; Awasthi et al., 2007; Gagliani et al., 2015).Tfh cells require IL-21, they express Bcl6 (Johnston et al., 2009; Nurieva et al., 2009; Yu et al., 2009; Crotty, 2014).

**Table 1:** CD4+ T cell types, their associated transcription factors, characteristic cytokines, and exogenous cytokines that induce the cell type.

Cell type	Transcription factor	Characteristic cytokines	Induced by
Th1	T-bet	IFNγ	IFNγ, IL-12
Th2	GATA3	IL-4	IL-4, IL-2
Th17	RORγt	IL-17, IL-21	TGFβ, IL-6, IL-21
Tfh	Bcl6	IL-21	IL-21
Th9	-	IL-9	TGFβ, IL-4
iTreg	Foxp3	TGFβ	TGFβ, IL-2
Tr1	-	IL-10	IL-10, IL-27
Th3	-	TGFβ	TGFβ


Furthermore, CD4+ T cells are highly heterogeneous suggesting that cell populations go through a continuum of polarization levels after initial priming (Murphy and Stockinger, 2010; Magombedze et al., 2013; DuPage and Bluestone, 2016; Eizenberg-Magar et al., 2017). Thus, mixed cellular phenotypes may be encountered under particular cytokine concentrations and combinations, and in some cases, hybrid cell types such as Th1-like and Th2-like regulatory cells or Th1/Th2 hybrids have been documented (Koch et al., 2009; Hegazy et al., 2010; Wohlfert et al., 2011). Studies performed on polarized CD4+ T cell populations indicate that, even under controlled *in vitro* conditions, stimulation generates heterogeneous cell populations with variable cytokine expression profiles or intermediate cell types (Assenmacher et al., 1994; Bucy et al., 1994; Openshaw et al., 1995; Kelso et al., 1999; Chang et al., 2007; Eizenberg-Magar et al., 2017). Asymmetric cell division with segregation of signaling proteins may explain this behavior (Verbist et al., 2016).

The same cytokines responsible for the induction of naïve cells to a particular polarized state may also dictate the conversion from a different subset to this state. For example, multiple studies report the transit of Treg cells toward Th17 cells in response to the addition of exogenous IL-6 in the presence of TGFβ (Yang et al., 2008; Lee et al., 2009a; Murphy and Stockinger, 2010). Other plastic transitions depend on the degree of polarization, as in the case of the Th17/Treg (Michalek et al., 2011; Berod et al., 2014; Gagliani et al., 2015) and the Th1/Th2 transition (Perez et al., 1995; Murphy et al., 1996; Hegazy et al., 2010). Recently polarized Th1 and Th2 cells can transdifferentiate into other subsets in response to environmental IL-4 or IL-12, but fully polarized Th1 and Th2 cells are robust and do not change their state in response to different microenvironments (Murphy et al., 1996). Despite abundant experimental data on such rich differentiation and plastic responses of CD4+ T cells in contrasting microenvironments, we still do not understand the underlying system-level mechanisms that explain such responses. To contribute in this direction our group and others have been integrating complex multistable regulatory network models that have been partially validated with experimental data (Mendoza, 2006; Naldi et al., 2010; Carbo et al., 2013; Abou-Jaoudé et al., 2014; Martinez-Sanchez et al., 2015; Eizenberg-Magar et al., 2017).

Complex regulatory networks are useful to model multistability, as they reach different stable multidimensional configurations, called attractors that correspond to expression profiles of different cell types (Kauffman, 1969; Mendoza et al., 1999; Bornholdt, 2008; Villarreal et al., 2012; Martínez-Sosa and Mendoza, 2013; Albert and Thakar, 2014; Naldi et al., 2015; Alvarez-Buylla et al., 2016). Hence, this type of models have been used in other systems to successfully explore the system-level mechanisms underlying cell differentiation (Kauffman, 1969; Mendoza et al., 1999; Bornholdt, 2008; Cortes et al., 2008; Azpeitia et al., 2011, 2014; Villarreal et al., 2012; Martínez-Sosa and Mendoza, 2013; Albert and Thakar, 2014; Naldi et al., 2015; Alvarez-Buylla et al., 2016; Davila-Velderraín et al., 2017). We previously proposed a Boolean network model that incorporates critical components to study CD4+ T cell subsets differentiation and plasticity (Martinez-Sanchez et al., 2015). In the present paper we have extended the Boolean model to a system with network interactions defined by fuzzy logic propositions. In this kind of approach, a fuzzy variable may acquire truth values within the continuous range [0,1]. The dynamic evolution of the network relations are described by a set of ordinary of differential equations (ODE) that enables us to analyze the role of alterations on cytokines concentrations and combinations, as well as other system’s components modifications on CD4+ T cell differentiation and plasticity. Each cell state or type corresponds to an attractor, and our system let us to study the conditions required to drive the system from one attractor to another one (Haken, 1977). We explore pathways that lead to equilibrium points, but also alterations of the expression levels of the networks components and the microenvironment, that may induce that cells transit between attractors (Mendoza, 2006; Naldi et al., 2010; Carbo et al., 2013; Abou-Jaoudé et al., 2014; Martinez-Sanchez et al., 2015; Eizenberg-Magar et al., 2017; Barberis et al., 2018; Puniya et al., 2018).

The continuous network model proposed here allows semi-quantitative evaluations of alterations of the inputs (exogenous cytokines) and the intrinsic components (transcription factors, signaling pathways, and autocrine cytokines) on cell-type transitions (Villarreal et al., 2012; Davila-Velderrain et al., 2015). The study involves an adaptation of a method specifically designed to study the so-called epigenetic landscape repatterning under altered microenvironmental conditions (Davila-Velderrain et al., 2015; Perez-Ruiz et al., 2015). Our model involves a set of regulatory interactions results that reproduce the main polarized phenotypes of CD4+ T cells and several of the plasticity patterns reported in the experimental literature. We determine the effect of systematic changes in the concentrations of exogenous cytokines and the internal state of the network in the differentiation and plasticity of CD4+ T cells. We focus on the Th1/Th2, and Th17/iTreg transitions, given that these have been thoroughly characterized, due to their pathogenic and therapeutic relevance (DuPage and Bluestone, 2016). This approach uncovers the signaling circuitry underlying the robust fully polarized Th1 and Th2 responses, and predicts that the phenotypic shift from a cell-mediated cytotoxic to a humoral immune response is possible only in early stages of CD4+ T cell differentiation. It also shows that a shift from inflammatory to induced regulatory immune response is much less restrictive. This finding and the overall framework put forward here may be useful to further understand the systemic mechanisms underlying immunological diseases where cellular plasticity plays a prime role (DuPage and Bluestone, 2016).

## Materials and Methods

### Network Construction

**FIGURE 1:**
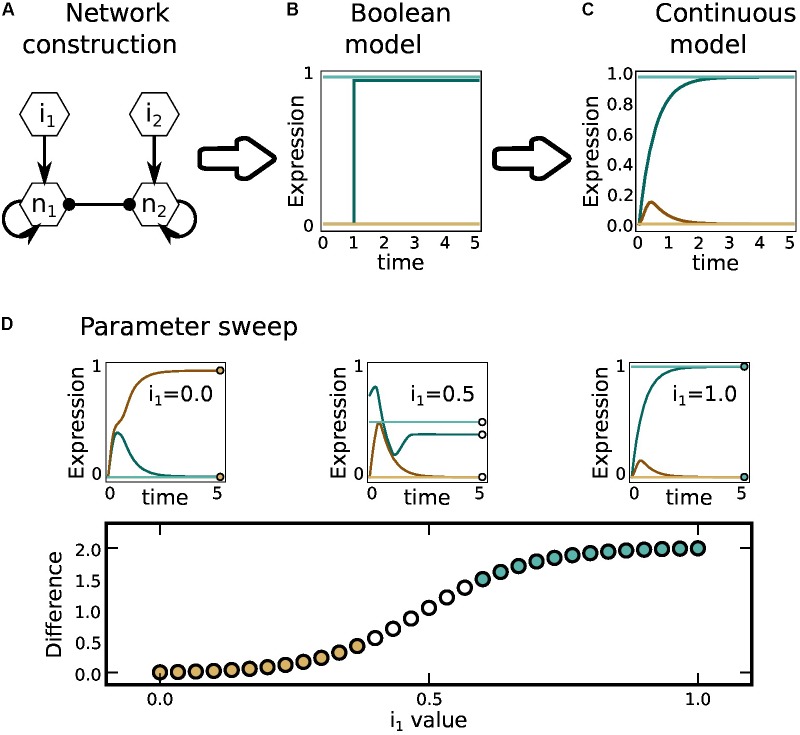
Methodology. Using available experimental data we constructed **(A)** the regulatory network, **(B)** Boolean functions (Martinez-Sanchez et al., 2015), and **(C)** ordinary differential equations (current article). **(D)** We then determined the resulting steady state for different concentrations and exogenous cytokines.

**FIGURE 2:**
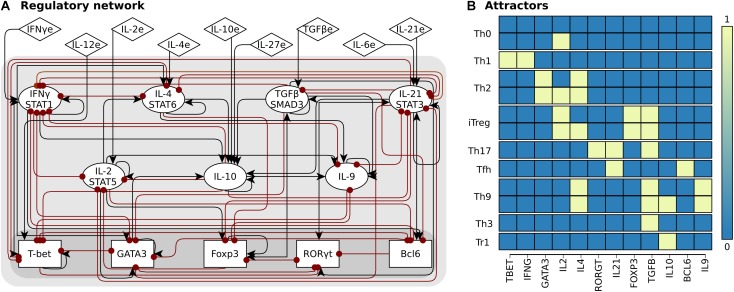
CD4+ T cell transcriptional-signaling regulatory network. The regulatory network was constructed using available experimental data. **(A)** The network includes transcription factors (rectangles), autocrine cytokines and their signaling pathways (ellipses) and exogenous cytokines (diamonds). Interactions leading to activation are represented by black arrows, while those leading to inhibition with red dots. **(B)** Sample attractors of the system.

We constructed the CD4+ T cell regulatory network using available experimental data (**Figure 1A**). The network includes nodes that correspond to transcription factors, signal transduction pathway components, and cytokine receptors, as well as autocrine and exogenous cytokines. The edges of the network correspond to the verified regulatory interactions between the nodes (**Supplementary Data Sheets S1, S2**) (Martinez-Sanchez et al., 2015). The value of the node depends on the state of its regulators defined by a logical rule (**Figure 1B**). In the Boolean approach, each node of the network has a value that corresponds to its expression level, where 0 corresponds to the basal level of expression (inactive) and 1 to the maximum normalized expression level (active), while in the continuous model the value of each node is a real number in the range [0,1]. The model was validated by verifying that the predicted CD4+ T cell subsets and plasticity transitions coincide with experimental observations (**Figure 2** and **Supplementary Data Sheet S2**) (Martinez-Sanchez et al., 2015).

The final network consists of 21 nodes (**Figure 2**). Five nodes correspond to transcription factors (TBET, GATA3, FOXP3, RORGT, and BCL6); seven nodes correspond to signaling pathways integrating signal transducers such as STAT proteins, interleukin receptors, and autocrine cytokines (IFNG, IL2, IL4, IL10, TGFB, IL9, and IL21); nine nodes correspond to exogenous cytokines, that are produced by other cells of the immune system and thus act as inputs to the network (IFNGe, IL12e, IL2e, IL4e, IL10e, IL27e, TGFBe, IL6e, and IL21e). These are marked with an “e” (exogenous) after the cytokine name. To study the effect of the microenvironment we focused on nine biologically relevant environments (Zhu et al., 2010): pro-Th0, pro-Th1, pro-Th2, pro-Th17, pro-Th9, pro-Tfh, pro-iTreg, pro-Tr1, and pro-Th3 (**Table 1**). The regulatory cytokine IL-10 deserves special consideration, since it uses STAT3, similarly as IL-2 and the inflammatory cytokines IL-6. Thus, we assume that IL-10 signaling is mediated by an independent pathway, different from that of IL-6/IL-21, even though they share STAT3 as a messenger molecule (Moore et al., 2001). While IL-27 has been linked to multiple functions, we consider that its main role in the model is regulatory (Awasthi et al., 2007; Murugaiyan et al., 2009; Pot et al., 2009). The model ignores weak interactions, chemokines, and epigenetic regulation that are also relevant and should be included in future modeling efforts.

### Fuzzy Logic Approach

The Boolean scheme allows to establish the main topological features of the network interactions; however, it only includes variables with dichotomous values. A more realistic approach should consider that variables and parameters with a continuous range of expression values. With that purpose we propose a model based on fuzzy logic where, not only the variables, but also the logical propositions describing the network relations are continuous. Fuzzy logic is aimed to provide formal foundation to approximate reasoning, including common language (Zadeh, 1965; Dubois et al., 1997; Novak et al., 1999). It is characterized by a graded approach, so that the degree to which an object exhibits a property is specified by a characteristic function (specified below) with truth values ranging between completely false (0, inhibited, or unexpressed), to completely true (1, activated, or expressed). The theory satisfies the axiomatics as Boolean logic, with the exception of the principles of no-contradiction, and the excluded middle. The first one states that a proposition and its negation may not be simultaneously true; the second that, for any proposition, either that proposition is true or its negation is true. Fuzzy logic has been applied in a number of engineering applications, such as control systems or pattern recognition.

The Boolean network interactions may be extended to the fuzzy realm by means of the following rules:

**Table:** 

*p and q*	*p⋅q*
*p or q*	*p + q-p⋅q*
*not p*	*1-p*

Since a proposition *w* and its negation 1*-w* may be simultaneously true, it follows that *w* = 1-*w* is a valid statement with solution *w_thr_* = *1/2* (Kosko, 1990). Thus, *w_thr_* is a threshold value between falsity and truth or, equivalently, between inhibited and active, a result which we employ below.

The regulatory network consists of *n* interacting nodes with expression levels at a time *t* given by *q_i_(t) (i = 1,…,v)*. The state of this node is regulated by its interaction with the rest of the network nodes, represented by a composite fuzzy proposition *w_i_(q_i_,…,q_N_)* that summarizes experimental observations. Following similar lines as those employed in logistic inference, it may be shown that the expression level of *w_i_* may be parameterized by a characteristic function with a logistic structure:

$$\Theta \left[w_{i}\right]=\frac{1}{1+\exp\left[-b\left(w_{i}-w_{thr}\right)\right]}$$

Here, the parameter *b* indicates the progression rate of *w_i_* from false to true, gradual for small *b*, sharp for large *b*. Since we are interested in representing input functions with a differentiable step-like behavior we employ *b = 25*. The model predictions do not depend upon specific choices of *b*, as long as this parameter is large enough (*b* ≥ 10) (**Supplementary Figure S1**).

### Continuous Dynamical Model

The dynamic evolution of the expression level *q_i_(t)* is driven by the regulatory network interactions described by the membership function 𝜃*[w_i_]*. The rate of change of *q_i_(t)* is thus determined by a set of ODEs (**Figure 1C** and **Supplementary Data Sheet S4**) of the form:

$$\frac{dq_{i}}{dt}=\Theta \left[w_{i}\right]-\alpha_{i}q_{i}$$

Here, *α_i_* is the decay rate of the expression of node *i*, so that in absencon level suffers an exponential time decay at a rate *α_i_*. In this paper we suppose that *α_i_= 1* for all nodes, so that the stationary expression level of node *i* is merely given by the degree of truth of the fuzzy proposition *w_i_*. The value of the parameter *α_i_* does affect the transitions of the system. However, a sensitivity analysis of this parameter is beyond the scope of this paper and it merits a separate paper, as can be seen in Davila-Velderrain et al., 2015.

The resulting attractors of the dynamical system are presented in **Supplementary Data Sheet S4**. They may be obtained as asymptotic states of the network dynamics i.e., by considering the limit *t*→∞ of the solutions. They satisfy the steady-state condition *dq_i_/dt = 0*, which leads to the expression

$$q_{i}^{ST}=\frac{1}{\alpha_{i}}\Theta \left[w_{i}\left(q_{1}^{ST},\ldots ,q_{n}^{ST}\right)\right]$$

Although it is not the purpose of the present work, the continuous fuzzy description may be easily extended to a stochastic regime by adding a noise variable ξ_i_(*t*) (with appropriate statistical properties) at the right hand side of the ODE system (see Di Cara et al., 2007; Wittmann et al., 2009; Villarreal et al., 2012).

### Polarization Analysis

The fuzzy logic model enabled evaluations of continuous alterations of the inputs (exogenous cytokines) and the intrinsic components (transcription factors, signaling pathways, and autocrine cytokines) of the network. To model polarization processes we studied the final steady states induced by stimulation associated to a specific cytokine environment on an initial Th0 state that corresponds to a CD4+ T cell under non-polarizing cytokine conditions. Dynamical simulations were performed for different sets of initial conditions and relative concentrations of microenvironmental cytokines to obtain the final steady states (**Figure 1D**). We considered that a node is actively expressed if its steady state value *q_i_ ≥ 0.75*, unexpressed if *q_i_ ≤ 0.25*, while intermediate values, *0.25 < q_i_ < 0.75*, correspond to a transition zone, with no definite expression. By using this criteria, it was stated that a steady state of the system corresponds to a CD4+ T cell subset if its corresponding transcription factors and cytokines are actively expressed, while states with null or low expression levels of all transcription factors were considered as Th0 (**Supplementary Data Sheet S5**).

Given the continuous nature of the regulatory network model presented here, it is impossible to determine all the possible steady states, since they are determined by an infinite set of initial conditions with expression values lying in the range [0,1]. We solved this problem by first verifying that the cell subtypes (or phenotypes) predicted by the discrete model are recovered in the continuous approach when the initial conditions are limited to the values 0 or 1; in that case, steady states stemming from the whole continuous range of initial conditions may be classified according to their similarity to cell types prognosticated by the Boolean model: Th0, Th1, Th2, Th17, Treg, Tfh, Th9, Tr1, and Th3 (**Supplementary Data Sheet S6**). It is understood that a continuous steady state is similar to Boolean state if its active nodes are coincident (with *q_i_ ≥ 0.75*). Steady states with intermediate expression values were considered to be in a transition zone (t.z.) of phenotypic coexistence.

### Plastic Transitions and Repatterning Analysis

**FIGURE 3:**
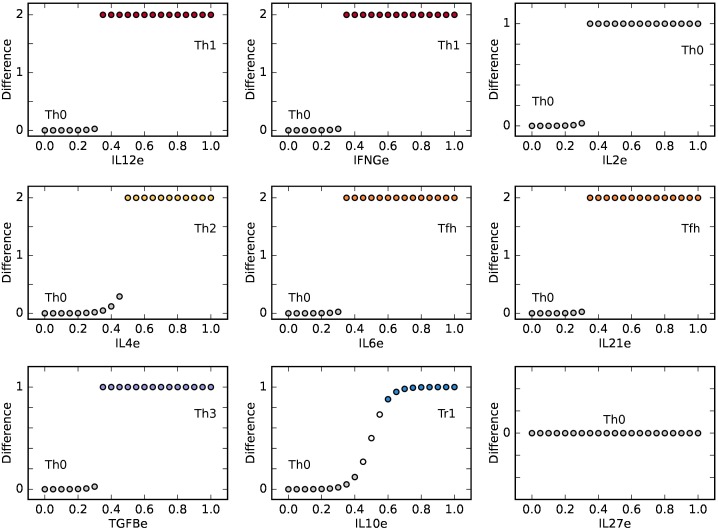
CD4+ T cell fate as a function of the concentration of single exogenous cytokines: IL12, IFNG, IL2, IL4, IL6, Il21, TGFB, IL10, and IL27. From an initial state TH0, a CD4+ T cell may acquire diverse phenotypes on an abrupt or gradual transition, depending on critical concentrations of environmental cytokines. The plot shows the difference between the values of the initial Th0 state and the final steady state at different concentrations of exogenous cytokines. We observe that the presence of either IL12 or IFNg is sufficient for Th1 polarization, as well as IL4, is sufficient for TH2 polarization. On the other hand, IL2 alone does not lead to an effector phenotype. Similarly, the presence of either IL6 or IL21 alone is sufficient for Tfh induction, as is the case of TGFB and IL10, leading to Th3 and Tr1, respectively. IL27 alone does not lead to any fate transition in this model.

In order to model plastic transitions, we considered a cell in an already partial or fully polarized state determined by different expression levels of the characteristic transcription factors and cytokines (**Figure 1D** and **Supplementary Data Sheet S3**), as defined before. In both kinds of simulations, we represented the effect of the microenvironment using a selected set of exogenous cytokines (**Table 1**) active at relative concentrations in the range *0 ≥ q_i_ ≥ 1*. Repatterning analyses were conducted numerically using an algorithm presented in Davila-Velderrain et al., 2015. A specific attractor was taken as an initial condition in an ODEs initial-value problem. For each active node in the attractor an ordered set of concentration values of exogenous cytokines was chosen, leaving constant the rest of system parameters. The ODEs were then solved numerically until reaching a steady state *q_i_^ST^*, each time using a slightly different exogenous cytokine concentration, and for all concentrations in the set. In order to identify bifurcating solutions of the ODE, a plot was generated for the total sum *Q* for the absolute value of the difference between the final and initial expression values of single-nodes

$$Q=\sum_{i=1}^{n}q_{i}^{ST}$$

as function of the varying expression value, as depicted in **Figures 2, 3**. Phenotypic transitions are distinguished by the occurrence of notorious jumps of the parameter *Q*, denoted as distance in the bifurcation graphs. The former method was employed to investigate reported CD4+ T cell phenotypic transitions induced by environmental cytokines with high immunological and pathogenic relevance like Th1/Th2 and Treg/Th17. The code for all the simulation experiments per- formed in this work is available in **Supplementary Data Sheet S7**.

## Results

### Effect of Exogenous Microenvironment on CD4+ T Cell Differentiation

To evaluate how altered concentrations of exogenous cytokines in the microenvironment shape CD4^+^ T cell differentiation, we studied the activation process of a Th0 cell as a function of increasing concentrations of the exogenous cytokines and determined the final steady states (**Figure 3**). We found that the exogenous cytokines IL12e, IFNGe, IL4e, IL6e, IL21e, TGFBe, and IL10e induce the differentiation from a Th0 initial steady state toward Th1, Th2, Tfh, Th3, and Tr1 subsets, respectively. Experimentally, these cytokines have been described as sufficient to induce differentiation into their associated cell types and are part of the feedback loops with the characteristic transcription factors of such types (Zhu et al., 2010). On the other hand, our model predicts that Th17, Th9, and iTreg subsets are not induced by alterations in a single exogenous cytokine in the micro-environment. Th17 cells requires exogenous TGFβ in addition to IL6e/IL21e, Treg cells require constant IL-2 in the microenviroment in addition to TGFβ and Th9 cells are highly dependent on the presence of both IL-4 and TGFβ (Zhu et al., 2010; Schmitt et al., 2014).

The critical concentration required to induce a transition varied depending on the particular exogenous cytokine being modified. IL12e, IL6e, and IL21e required relatively small concentrations (0.2) to induce the differentiation from Th0 to Th1 and Tfh, respectively, while IL4e required a higher concentration (0.36) to induce the differentiation from Th0 to Th2. On the other hand, IL2e and IL27e alone were not able to induce transitions. We observed that IL2e induced the expression of high levels of IL2; however, we labeled the resulting cells as Th0, as IL-2 production by itself is not associated with a particular polarization subset.

It is also interesting to note that transitions among subsets have different patterns of sensitivity to exogenous cytokine concentrations. Most of the transitions from Th0 to other subsets were discontinuous; once a threshold concentration was achieved, the cell changed its expression pattern to a different one in an abrupt manner. An exception was observed when IL10 was used as an inducer. This cytokine caused a gradual transition from Th0 to Tr1; in this case, a continuous range of steady states was recovered in the transition zone between both subsets. These results predict that, for most of single cytokines, CD4^+^ T cells should initiate differentiation once the threshold concentration has been reached, whereas these cells may display a range of sensitivities to altered concentrations of other cytokines in order to switch to a different state or phenotype.

**FIGURE 4:**
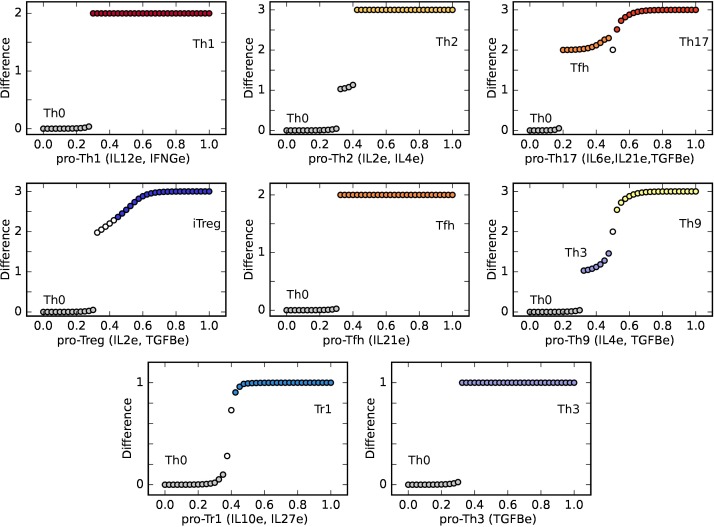
T-CD4 cell fate as a function of exogenous cytokine concentrations define diverse phenotype-associated environments. From the Th0 initial state, a CD4+ T cell evolves to different phenotypes, depending on critical concentrations of environmental cytokines as shown in **Table 1**: Th1 (IFNG and IL12), Th2 (IL4, Il2), Th17 (Il21, TGFB), Treg (IL2, TGFB), Tfh (IL21), Th9 (IL4, TGFB), Tr1 (IL10, IL27), and Th3 (TGFB). The plot shows the difference between the values of the initial Th0 state and the final steady state at different concentrations of exogenous cytokines. The transition may be abrupt or gradual and, interestingly, may involve an intermediate state, as in the cases Th0 - > Tfh - > Th17 (C), and Th0 - > Th3 - > Th9 (F).

CD4^+^ T subsets such as Th9, Th17, and iTreg require particular combinations of cytokines to differentiate from naïve cells. In our model, we simulated the activation of a Th0 cell in the presence of different combinations and concentrations of the exogenous cytokines associated with the microenvironment (**Table 2** and **Figure 4**). In the case of requiring more than one exogenous cytokine, all the implicated nodes were set to the same value. Using this methodology, we were able to induce the differentiation from a Th0 steady state toward Th1, Th2, Th17, Th9, Tfh, iTreg, Th3, and Tr1 subsets by cytokine combinations that are in agreement with experimental data (Zhu et al., 2010; Crotty, 2014; DuPage and Bluestone, 2016).

**Table 2:** Exogenous cytokines in different environments included in the CD4+ T cell regulatory network.

Micro-environment	Active input nodes
pro-Th0	None
pro-Th1	IFNGe, IL12e
pro-Th2	IL2e, IL4e
pro-Th17	IL21e, TGFBe
proTh9	IL4e, TGFBe
proTfh,	IL21e
pro-iTreg	IL2e, TGFBe
pro-Tr1	IL10e, IL27e
pro-Th3	TGFBe


*Active nodes refer to the same exogenous cytokines, whose concentrations were modified during the simulation, adopting values between 0 and 1.*

The concentration required to induce polarization when using multiple cytokines varied depending on the CD4^+^ T initial cell type. Under their combined action the individual concentrations are lower (**Figure 4**) than those required in the case of a single exogenous cytokine (**Figure 3**). This result suggests that the regulatory network mediates a synergistic effect of cytokines on CD4^+^ T cell differentiation. For example, while a concentration of IL4e = 0.36 was necessary to induce the polarization toward Th2, a concentration of IL 2e and IL4e = 0.26 was sufficient to induce the same effect. Similarly, while a concentration of IL10e = 0.6 was necessary to induce the polarization toward Tr1, a concentration of IL10e and IL27e = 0.43 produced the same transition. Furthermore, autocrine IL10 achieved its maximum value with a lower concentration of exogenous cytokines when IL10e and IL27e act synergistically.

**Figure 4** shows that differentiation processes in pro-Th1, pro-Th2, and pro-Tfh microenvironments were abrupt, while the transition in a pro-Tr1 environment was gradual. In a pro-Th17, pro-Th9, and pro-iTreg alterations in the micro-environments, including TGFβe, caused a small abrupt change followed by a gradual change in the expression levels of the components in the steady state configuration. In the pro-Th17 and pro-Th9 the model predicted an intermediate step before the final polarized state was achieved. In the pro-Th17 case, increasing cytokine levels induced an initial abrupt change toward a plateau zone corresponding to Tfh, followed by a transition to the Th17 steady state. A similar behavior was observed in the pro-Th9 microenvironment with a precursor TGFβ ^+^ (Th3) subset, followed by a final Th9 steady state. It is worth noting that TGFβ has a key role in the induction of the three types of CD4^+^ T cell types discussed here and it has complex interactions with other exogenous cytokines in their effects on cell plasticity (Eizenberg-Magar et al., 2017). These results illustrate that the continuous versión of our minimal CD4+ T cell differentiation model comprises a useful working hypothesis concerning the dynamic and complex mechanisms underlying how the microenvironment alters cell plasticity in response to TGFβ in the immune system.

In summary, the continuous model presented in this paper recovers CD4+ T cell plasticity responses to cytokine concentrations that have been documented experimentally and explains how such patterns of cell-type shifts depend on the initial CD4+ T cell type, being sometimes abrupt and others gradual. It also shows that cytokine combinations and, notably, the induction of different subsets under the action of different concentrations of the same cytokine combinations underlie different patterns of CD4+ T cell transitions.

### Effects of the Exogenous and Endogenous Microenvironment on CD4^+^ T Cell Plasticity

**FIGURE 5:**
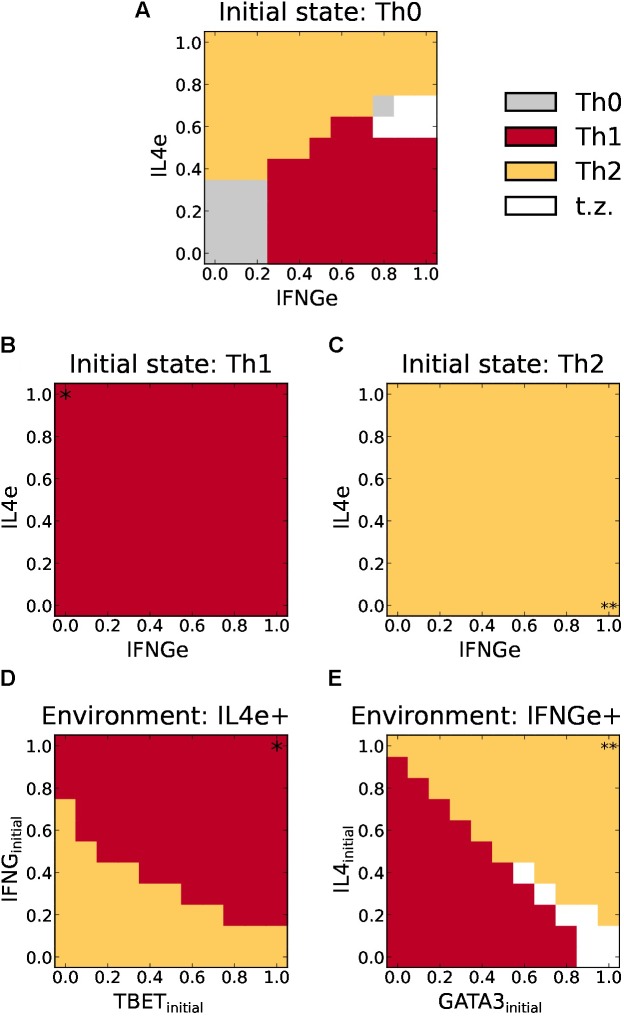
Phenotype space diagrams for Th1 and Th2 polarization and plasticity as a function of the relative concentration of environmental IFNg and IL4, and expression of transcription factors. **(A)** Diagram for cell differentiation assuming an initial Th0 state. As the external concentration of IFNGe increases, the system develops an abrupt transition from Th0 to Th1. Similarly, an increase in external IL4e drives an abrupt transition from TH0 to Th2. For moderate concentrations of IFNGe and IL4e (< 0.8), we observe two wide zones of Th1 or Th2 prevalence with a sharp boundary, meaning that small variation of cytokines at these zones may change cell polarization. A transition zone with no defined polarization appears at higher concentrations of these cytokines (white and gray areas). **(B,C)** Plasticity diagrams assuming full Th1 **(B)** or Th2 **(C)** polarized states (i.e., induced by INFg = 1 and IL-4 = 1 in diagram **A**, respectively). No phenotypic transitions are observed under variable concentrations of environmental IL4e and autocrine IFNGe. **(D)** Plasticity diagram of Th1 cells assuming an environmental concentration of IL4e = 1. Cells require the production of initial high levels of autocrine IFNG and expression of TBET to maintain a Th1 phenotype. If the initial expression levels decrease, especially in the case of autocrine IFNG, it will transit into a Th2 cell. **(E)** Plasticity diagram of Th2 cells assuming an environmental concentration of IFNGe = 1. The cell requires the production of high levels of autocrine IL4 and expression of GATA3 to maintain a Th2 phenotype. If the initial expression levels decrease it will transit into a Th1 cell. At high expression levels of initial GATA3 and low initial IL4, there exists a transition zone where the cell cannot be classified.

We first focus on the transition between Th1 and Th2, that has been experimentally observed, particularly when these cells have recently differentiated, but not when they are fully polarized (Perez et al., 1995; Panzer et al., 2012). To study this process we considered the response of already differentiated Th1 and Th2 states, in response to variable concentrations of a defined cytokine for a particular subset, in combination with the opposing cytokine (IFNGe for Th2, and IL4e, for Th1), and then we used the model to predict the final steady state. **Figure 5** shows that when the initial configuration of the system corresponded to a highly polarized Th1 (TBET and IFNG = 1) or Th2 (GATA3 and IL4 = 1) states, for every combination of (exogenous) IL4e and IFNGe concentrations, the system remained in its original state even under high concentrations of all these cytokines. This, indicates that highly polarized Th1 or Th2 cells are not plastic. However, by considering initial lower concentrations of Th1 and Th2 transcription factors and cytokines, consistent with partial phenotype polarization, plastic transitions are predicted by the model. CD4^+^ T cells require the production of high levels of autocrine IFNG and expression of TBET to maintain a Th1 phenotype. If the expression levels decrease, especially in the case of autocrine IFNG, Th1 cells are predicted to transit into Th2 cells. At the same time, the Th2 cells require the production of high levels of autocrine IL4 and expression of GATA3 to maintain a Th2 phenotype. If the initial expression levels decrease these cells are expected to transit to Th1 cells. At high initial levels of GATA3 and low IL4, a transition zone at which cells display mixed characteristics is predicted. These results show that plasticity between the Th1 and Th2 subsets depends not only on the microenvironment cytokines, but also on the intracellular state.

**FIGURE 6:**
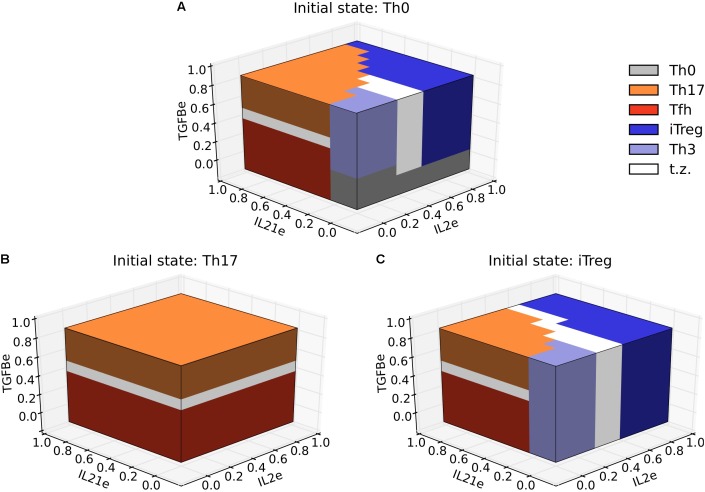
Three-dimensional phenotype space diagrams for Th17 and iTreg polarization and plasticity as a function of the relative concentrations of IL2, IL21, and TGFB in the microenvironment. In the differentiation diagram **(A)** we observe alternative phenotypic regions defined by relative concentrations of environmental cytokines. The regions may be either separated by a sharp boundary or by a more gradual transition zone (labeled in white). The plasticity diagram **(B)** indicates a polarized behavior for Th17 versus Tfh phenotype determined by a high or low concentration of external TGFB. A richer behavior ensues when the initial state is Treg, as shown in the plasticity diagram **(C)**. We observe a similar structure as that depicted in **A**, except that the Th0 zone is absent.

The transition between Th17 and iTreg, has been extensively investigated experimentally (Xu et al., 2007; Wei et al., 2008; Lee et al., 2009a,b; Littman and Rudensky, 2010; Kleinewietfeld and Hafler, 2013; Noack and Miossec, 2014) and is particularly important for some pathological conditions, such as chronic inflammation. To study this process we considered fully differentiated Th17 (RORGT and IL21 = 1) and iTreg cells (FOXP3 and TGFB = 1) under the presence of different concentrations of the exogenous cytokines, IL2e, IL21e, and TGFBe. In the case of Th17 cells, they remained in a Th17 phenotype at a high concentration of TGFBe, while they switched toward Tfh for lower concentrations of TGFBe (< 0.6). Some experiments have reported that induction of Th17 require exogenous TGFB (Veldhoen et al., 2006), but it is uncertain if the transition toward Tfh associated to low TGFB levels will occur in all cases. On the other hand, iTreg cells remain stable under high concentrations of IL2e, while they transit toward Th17, Tfh, or Th3 at low concentrations of IL2e (< 0.65) (**Figure 6**). These results show that plastic transitions between subsets are not symmetrical, and depend on the previous polarization state of the cell.

## Discussion

Our simulations show contrasting differentiation patterns of CD4+ T cells under different concentrations and combinations of exogenous cytokines, highlighting the importance of synergy and competing interactions among microenvironment components and CD4+ T cell network components to induce different patterns of CD4+ T cell plasticity. We also showed that plasticity between the Th1/Th2 and iTreg/Th17 subsets depends on varying the concentration of microenvironment cytokines and the expression level of intracellular transcription factors and autocrine cytokines depending on the initial cell type.

The model predicts both abrupt and gradual transitions between cell types. In abrupt transitions, there is a sudden change from an initial to a final steady state or cell type, once the concentration of exogenous cytokines exceeds a threshold value. This behavior suggests that the transition between stable cell phenotypes is energetically favorable once the threshold value has been achieved. In this process, exogenous cytokines provide the initial stimulus to promote the expression of both transcription factors and autocrine cytokines characteristic of a cell type that is different to the original one, while positive feedback loops greatly increase their polarization efficiency.

In contrast, in gradual transitions, steady states that express intermediate levels of transcription factors and autocrine cytokines appear. In these steady states, a clear-cut threshold between the two expression patterns is not observed, so they cannot be easily classified into one subset or another, signaling the manifestation of partially polarized states. The heterogeneity of CD4+ T cells has been well-documented (Murphy and Stockinger, 2010; DuPage and Bluestone, 2016; Eizenberg-Magar et al., 2017), and could be the result of regulatory circuits capable of generating a range of cells that express intermediate levels of specific molecules that can stably coexist or change from one another under certain conditions. It is important to notice that every gradual transition involves regulatory circuits with central nodes which display feedback interactions. Such feedback loops render stability to the initial polarization state so that its intrinsic cytokine production and transcription factor expression should gradually decrease under changing microenvironmental conditions. We observed this behavior especially in response to changes in the concentration of IL-10 and TGFβ. IL-10 is a regulatory cytokine produced by multiple CD4+ T subsets (Howes et al., 2014; Gagliani et al., 2015). TGFβ may display both regulatory and inflammatory effects and it is implied in the differentiation of multiple subsets like Th17, iTreg, and Th9 (Chen et al., 2003; Veldhoen et al., 2006; Davidson et al., 2007; Kaplan, 2013). It is conceivable that gradual transitions and generation of intermediate polarization states reflect the intricate regulatory signaling effects of TGFβ and of IL-21, and are probably responsible for tuning the effects of different conditions in the immune response (Grossman and Paul, 2015).

The model also captures some cases where there is an abrupt transition followed by a gradual transition in polarization processes. Such is the case of the Th0-Tfh-Th17, the Th0-Th3(TGFB+)-Th9 and the Th0-iTreg transitions. Interestingly, in all these cases TGFβ is present in the micro-environment. This indicates that the concentration of TGFβ may modulate the immune response in complex ways. These interesting results suggest a system-level explanation of previous experimental results. For example, it is known that TGFβ regulates Th17 cells in a differential way depending on the concentration and combinations of cytokines in the microenvironment (Yang et al., 2008). Furthermore, consistent with our simulations, it is known that the TGFβ signaling pathway is highly modulated (Attisano and Wrana, 2002; Travis and Sheppard, 2014). Our model also predicts that TGFβ may induce distinct subsets at different concentrations, in particular, Tfh, Th9, iTreg, and Th3. A careful analysis of this kind of regulatory circuits will shed light on the specific mechanisms defining transcriptional programs that lead to cell heterogeneity. Understanding the interactions underlying the dynamical behavior of T helper cells may help elucidate the regulatory role of this important molecule in the immune response.

The model presented in this paper also highlights the cooperation among different exogenous cytokines during differentiation. Th17, iTreg, and Th9 subsets require TGFβ in combination with IL-6/IL-21, IL-2, and IL-4 to differentiate, respectively, in agreement with experimental data (Chen et al., 2003; Veldhoen et al., 2006; Davidson et al., 2007; Kaplan, 2013). In other cases, the effect of a single cytokine is sufficient to induce polarization, but the synergy with other cytokines lowers the threshold concentration necessary to induce polarization. In this way, the model allows us to study and predict synergic relations among cytokines in CD4+ T cell differentiation.

As mentioned above, we also use the model to study the effect of opposing cytokines in differentiation and plasticity of Th1/Th2 and Th17/iTreg subsets. The Th1 and Th2 cells are highly stable, and the transition between them is hard to achieve experimentally (Perez et al., 1995; Murphy et al., 1996; Hegazy et al., 2010). Coincidently our model shows that, once these types have achieved a stable state, Th1 and Th2 are robust to changes in their microenvironment. This behavior seems consistent with a particularly robust interaction circuit, defined by coupled regulatory switching modules between mutually inhibitory nodes with negative feedbacks, each node defining an alternative regulatory route. However, partially polarized cells can transit to the other cell types when they are subject to an opposing cytokine (IL-4 in the case of Th1 or IFNγ in the case of Th2). In conclusion, our model provides a system-level mechanistic explanation to these complex behaviors of Th1 and Th2 cells.

The model also recovers the spontaneous transition of iTreg into Th17 in the presence of IL-21 or the closely similar IL-6 (here considered as equivalents) (Xu et al., 2007) at low concentrations of IL-2. The plasticity of this transition is not symmetrical, as changes in the microenvironment are not enough for Th17 to transit toward iTreg. For such transition, it is also necessary to alter the internal state of the cell, changing the expression levels of key transcription factors, as it has been shown in experimental studies (Michalek et al., 2011; Berod et al., 2014; Gagliani et al., 2015). These results seem to imply that the basin of attraction of iTreg is shallower than that of Th17. This could be the result of the different regulatory circuits implied in the differentiation of each cell type, since while both depend on TGFβ, iTreg both require and inhibit the production of IL-2 (Fontenot et al., 2003; Pandiyan et al., 2007), restricting the stability of these cells.

The model and simulations presented here are able to describe cell type transitions and the recovered patterns do not rely upon specific parameter estimates, but rather on the network structure and overall dynamic behavior. However, the exact transition points may change depending on the precise concentrations and parameters of the biological system (Eizenberg-Magar et al., 2017). Given the relative nature of the semi-quantitative variations introduced in the model, we should be cautious in providing precise quantitative predictions concerning the sensitivity of the different subsets under real experimental conditions. Theoretical models like the one presented here provide an ideal tool to integrate recent advances in experimental knowledge and provide a system-level mechanistic explanation for observed behaviors in experiments, and also to provide informed predictions for future experiments. Hence, the feedback between experimental and theoretical research is necessary to understand the rich behavior of CD4+ T cells and the immunological system.

## Conclusion

The continuous model with fuzzy logic interaction rules, presented in this paper, recovers CD4+ T cell plasticity responses to cytokine concentrations that have been documented experimentally and explains how such patterns of cell-type shifts results from feedback between initial T cell type and the microenvironment, being sometimes abrupt and others gradual. The simulations show how different cytokine combinations and, notably, the induction of different subsets under the action of different concentrations of the same cytokine combinations underlie different patterns of T cell transitions. The semi-quantitative nature of the model allows predictions that do not depend on specific parameters for which we are still lacking experimental support. This model may contribute to the study of immunological diseases where cellular plasticity is a key, such as cancer, and autoimmune diseases like type 1 diabetes, multiple sclerosis, or juvenile arthritis (DuPage and Bluestone, 2016).

## Author Contributions

EA-B and CVL conceived, planned, and coordinated the study. CVL and MM-S established the continuous model and performed simulations and calculations. LH contributed with her expertise on T cell signaling and immunological consequences. All authors participated in the interpretation, analyses of results, and wrote the paper.

## Conflict of Interest Statement

The authors declare that the research was conducted in the absence of any commercial or financial relationships that could be construed as a potential conflict of interest.

## Supplementary Material

FIGURE S1 Sensitivity analysis of the parameter *b*. Effect of various values of *b* (5, 10, 25, and 50) in abrupt (IL2e), gradual (IL10e), and mixed (IL6e + IL21e + TGFBe) transitions. The model predictions do not depend upon the specific choice of b if this parameter is large enough (*b* ≥ 10).

DATA SHEET S1 References of the CD4+ T cell regulatory network.

DATA SHEET S2 Boolean rules of the CD4+ T cell regulatory network.

DATA SHEET S3 Boolean attractors of the CD4+ T cell regulatory network.

DATA SHEET S4 Ordinary differential model equations of the CD4+ T cell regulatory network.

DATA SHEET S5 Boolean rules for labelling the attractors of the CD4+ T cell regulatory network.

DATA SHEET S6 Continuous attractors of the CD4+ T cell regulatory network.

DATA SHEET S7 Code and simulations of the CD4+ T cell regulatory network.
