# Supplementary figures and images for: Role of Cytokine Combinations on CD4+ T Cell Differentiation, Partial Polarization, and Plasticity: Continuous Network Modeling Approach
Source: Front Physiol. 2018 Aug 2;9:877. doi: 10.3389/fphys.2018.00877 (PMC6089340; doi:10.3389/fphys.2018.00877)

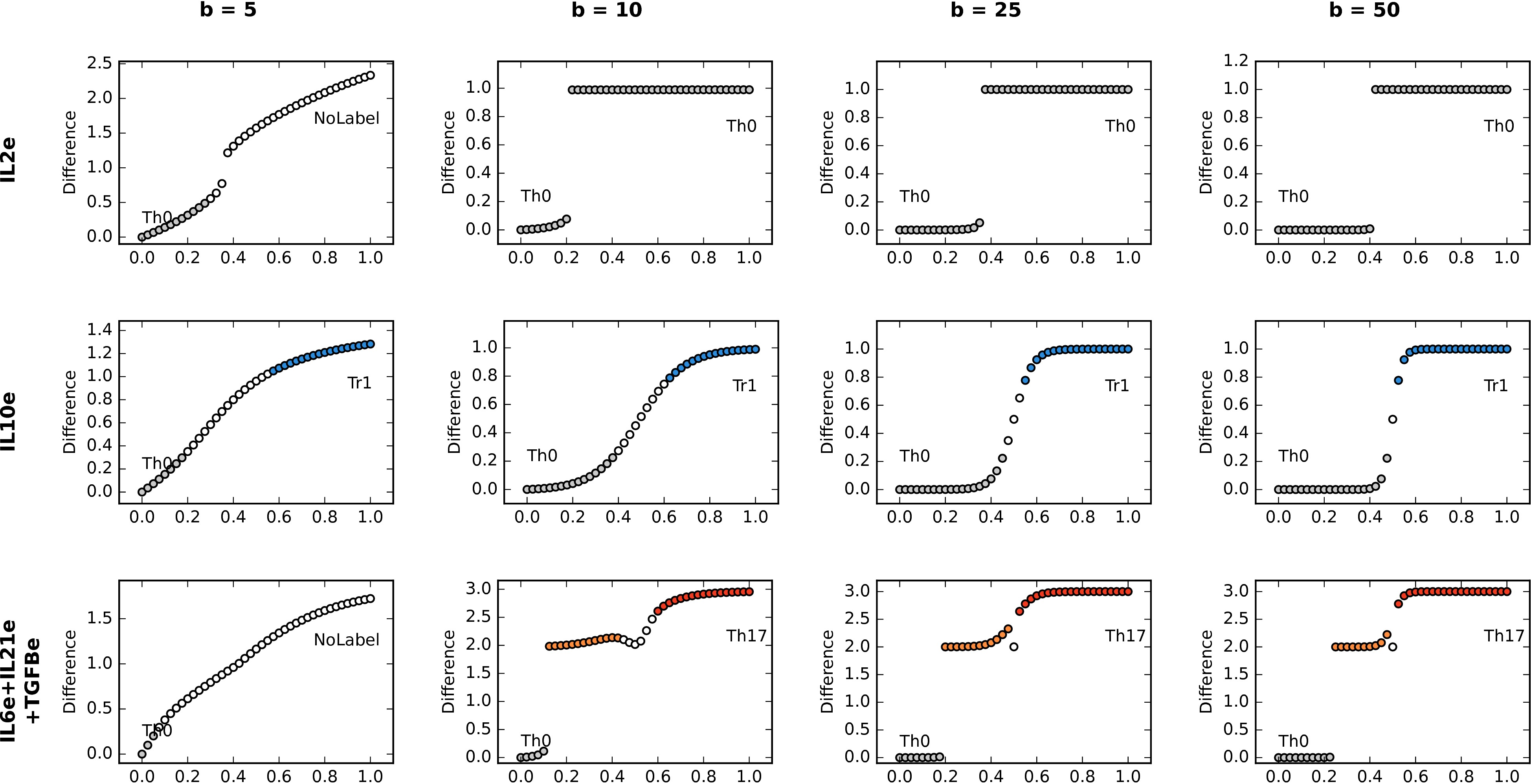

Supplement: FIGURE S1 — Sensitivity analysis of the parameter b. Effect of various values of b (5, 10, 25, and 50) in abrupt (IL2e), gradual (IL10e), and mixed (IL6e + IL21e + TGFBe) transitions. The model predictions do not depend upon the specific choice of b if this parameter is large enough (b ≥ 10). [file Image_1.JPEG]

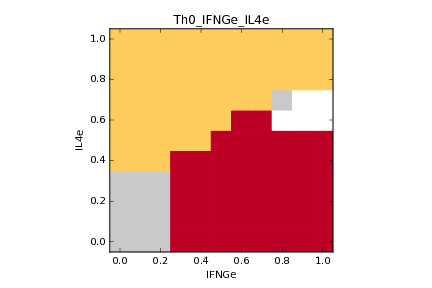

Supplement: DATA SHEET S7 — Code and simulations of the CD4+ T cell regulatory network. [file Data_Sheet_7.ZIP › plasticity-Nnodes/svg/Th1Th2/Th0_IFNGe_IL4e-mesh.png]

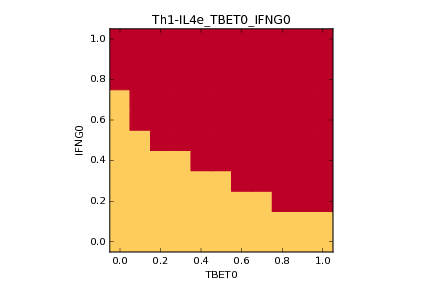

Supplement: DATA SHEET S7 — Code and simulations of the CD4+ T cell regulatory network. [file Data_Sheet_7.ZIP › plasticity-Nnodes/svg/Th1Th2/Th1-IL4e_TBET0_IFNG0-mesh.png]

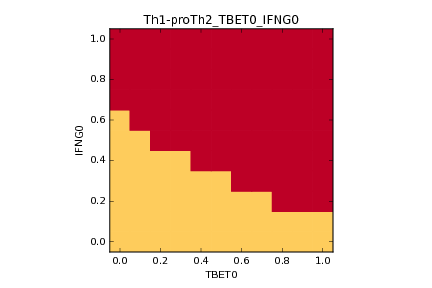

Supplement: DATA SHEET S7 — Code and simulations of the CD4+ T cell regulatory network. [file Data_Sheet_7.ZIP › plasticity-Nnodes/svg/Th1Th2/Th1-proTh2_TBET0_IFNG0-mesh.png]

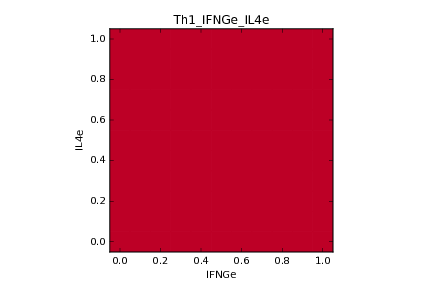

Supplement: DATA SHEET S7 — Code and simulations of the CD4+ T cell regulatory network. [file Data_Sheet_7.ZIP › plasticity-Nnodes/svg/Th1Th2/Th1_IFNGe_IL4e-mesh.png]

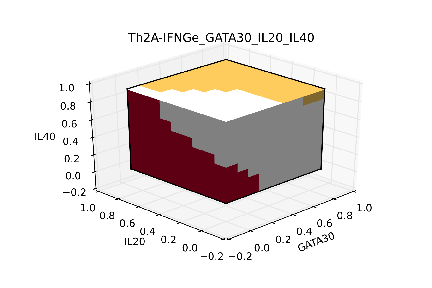

Supplement: DATA SHEET S7 — Code and simulations of the CD4+ T cell regulatory network. [file Data_Sheet_7.ZIP › plasticity-Nnodes/svg/Th1Th2/Th2A-IFNGe_GATA30_IL20_IL40-mesh.png]

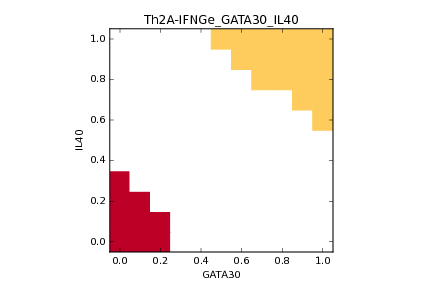

Supplement: DATA SHEET S7 — Code and simulations of the CD4+ T cell regulatory network. [file Data_Sheet_7.ZIP › plasticity-Nnodes/svg/Th1Th2/Th2A-IFNGe_GATA30_IL40-mesh.png]

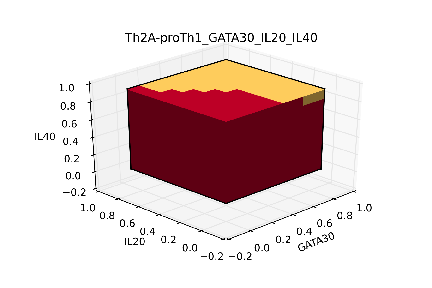

Supplement: DATA SHEET S7 — Code and simulations of the CD4+ T cell regulatory network. [file Data_Sheet_7.ZIP › plasticity-Nnodes/svg/Th1Th2/Th2A-proTh1_GATA30_IL20_IL40-mesh.png]

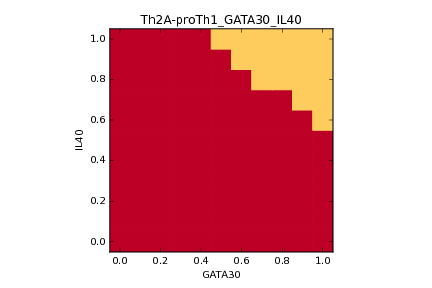

Supplement: DATA SHEET S7 — Code and simulations of the CD4+ T cell regulatory network. [file Data_Sheet_7.ZIP › plasticity-Nnodes/svg/Th1Th2/Th2A-proTh1_GATA30_IL40-mesh.png]

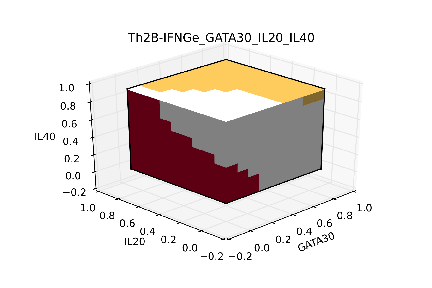

Supplement: DATA SHEET S7 — Code and simulations of the CD4+ T cell regulatory network. [file Data_Sheet_7.ZIP › plasticity-Nnodes/svg/Th1Th2/Th2B-IFNGe_GATA30_IL20_IL40-mesh.png]

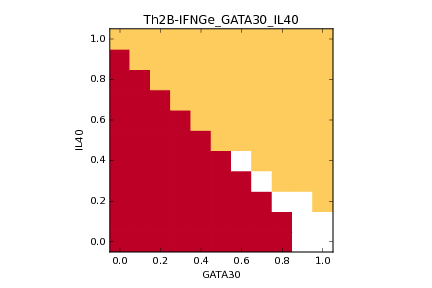

Supplement: DATA SHEET S7 — Code and simulations of the CD4+ T cell regulatory network. [file Data_Sheet_7.ZIP › plasticity-Nnodes/svg/Th1Th2/Th2B-IFNGe_GATA30_IL40-mesh.png]

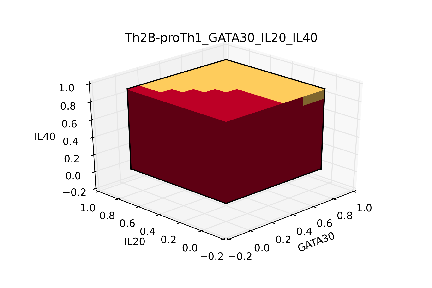

Supplement: DATA SHEET S7 — Code and simulations of the CD4+ T cell regulatory network. [file Data_Sheet_7.ZIP › plasticity-Nnodes/svg/Th1Th2/Th2B-proTh1_GATA30_IL20_IL40-mesh.png]

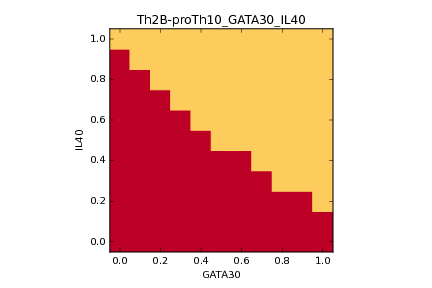

Supplement: DATA SHEET S7 — Code and simulations of the CD4+ T cell regulatory network. [file Data_Sheet_7.ZIP › plasticity-Nnodes/svg/Th1Th2/Th2B-proTh1_GATA30_IL40-mesh.png]

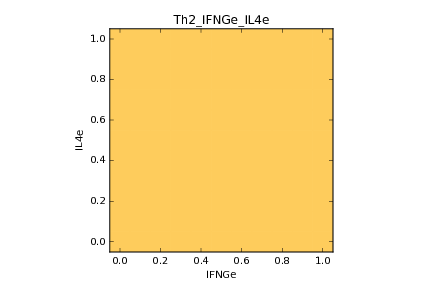

Supplement: DATA SHEET S7 — Code and simulations of the CD4+ T cell regulatory network. [file Data_Sheet_7.ZIP › plasticity-Nnodes/svg/Th1Th2/Th2B_IFNGe_IL4e-mesh.png]
